# Causes and age of neonatal death and associations with maternal and newborn care characteristics in Nepal: a verbal autopsy study

**DOI:** 10.1186/s13690-021-00771-5

**Published:** 2022-01-11

**Authors:** Daniel J. Erchick, Johanna B. Lackner, Luke C. Mullany, Nitin N. Bhandari, Purusotam R. Shedain, Sirjana Khanal, Jyoti R. Dhakwa, Joanne Katz

**Affiliations:** 1 Department of International Health, Johns Hopkins Bloomberg School of Public Health, 615 N. Wolfe Street, Baltimore, MD 21205 USA; 2 Avant Medical Communications Group, Aptos, CA USA; 3 Integrated Rural Health Development Training Centre, Maharajgunj, Kathmandu Nepal

**Keywords:** Nepal, Newborn, Neonatal, Verbal autopsy, Mortality

## Abstract

**Background:**

In Nepal, neonatal mortality fell substantially between 2000 and 2018, decreasing 50% from 40 to 20 deaths per 1,000 live births. Nepal’s success has been attributed to a decreasing total fertility rate, improvements in female education, increases in coverage of skilled care at birth, and community-based child survival interventions.

**Methods:**

A verbal autopsy study, led by the Integrated Rural Health Development Training Centre (IRHDTC), conducted interviews for 338 neonatal deaths across six districts in Nepal between April 2012 and April 2013. We conducted a secondary analysis of verbal autopsy data to understand how cause and age of neonatal death are related to health behaviors, care seeking practices, and coverage of essential services in Nepal.

**Results:**

Sepsis was the leading cause of neonatal death (n=159/338, 47.0%), followed by birth asphyxia (n=56/338, 16.6%), preterm birth (n=45/338, 13.3%), and low birth weight (n=17/338, 5.0%). Neonatal deaths occurred primarily on the first day of life (27.2%) and between days 1 and 6 (64.8%) of life. Risk of death due birth asphyxia relative to sepsis was higher among mothers who were nulligravida, had <4 antenatal care visits, and had a multiple birth; risk of death due to prematurity relative to sepsis was lower for women who made ≥1 delivery preparation and higher for women with a multiple birth.

**Conclusions:**

Our findings suggest cause and age of death distributions typically associated with high mortality settings. Increased coverage of preventive antenatal care interventions and counseling are critically needed. Delays in care seeking for newborn illness and quality of care around the time of delivery and for sick newborns are important points of intervention with potential to reduce deaths, particularly for birth asphyxia and sepsis, which remain common in this population.

**Supplementary Information:**

The online version contains supplementary material available at 10.1186/s13690-021-00771-5.

## Background

In Nepal, mortality among children younger than five years old decreased by 61% from 81 to 32 deaths per 1000 live births between 2000 and 2018 [1]. Among newborns, mortality also declined, but at a slower pace, falling 50% from 40 to 20 deaths per 1000 live births [1]. Nepal’s success has been attributed to a decreasing total fertility rate, improvements in female education, increases in coverage of skilled care at birth, and community-based child survival interventions [2, 3]. Achieving the Sustainable Development Goal (SDG) target of reducing neonatal mortality to 12 deaths per 1000 live births by 2030 will require an understanding, across the varied geographical regions in Nepal, of the causes of neonatal death, coverage of critical interventions for newborn survival, newborn care practices, and care seeking behaviors during newborn illness [4].

In 2007, Nepal’s Ministry of Health and Population (MoHP), guided by the National Neonatal Health Strategy 2004, developed the Community-Based Newborn Care Package (CB-NCP), a program aimed to deliver evidence-based health interventions targeting leading causes of neonatal mortality [5–7]. CB-NCP utilized facility-based and community-based health workers, including Female Community Health Volunteers (FCHV), who live in the communities where they work, for health promotion activities and delivery of community-based preventive and therapeutic interventions [5]. The program focused on seven areas: behavior change communication for birth preparedness and newborn care; promotion of institutional delivery or clean delivery practices in the home; recognition and management of birth asphyxia; postnatal care; care of low birth weight newborns; prevention and management of hypothermia; and management of newborn infection [5]. After the program was piloted in ten districts (2009–2010), it was scaled up across Nepal, and eventually merged with the Community-Based Integrated Management of Childhood Illness (CB-IMCI) in 2015 to create a unified approach (CB-IMNCI) to child survival [8, 9].

Understanding how neonatal mortality and morbidity are related to coverage of health interventions promoted by CB-IMNCI, as well as care seeking behaviors and management of newborn illness, are critical to improving survival for mothers and their babies [10–12]. Verbal autopsy is a widely-used methodology for determining cause of death in populations without a complete vital registration system [13]. Such surveys include questions about the clinical signs and symptoms, health behaviors and care seeking, and increasingly, social factors that impact access to care and health outcomes [14]. We conducted a secondary data analysis of data collected through a verbal autopsy study in six districts in Nepal between 2012 and 2013 to better understand how these factors were related to cause and age of newborn death.

## Methods

### Study design

This study was a descriptive secondary analysis of the associations between two outcomes – causes of death and age of death – and participant factors, including maternal characteristics, health care service utilization, and care seeking behaviors for neonatal deaths collected from six districts in Nepal between April 13, 2012, and April 13, 2013 (Nepali fiscal year 2069/2070). The original study was designed and implemented by a non-profit organization, the Integrated Rural Health Development Training Centre (IRHDTC), based in Kathmandu, Nepal, in collaboration with the Child Health Division, MoHP [15].

As lead of the original study, IRHDTC constructed the study sample with the aim of including all neonatal deaths in Dolpa, Jumla, Morang, Chitwan, Palpa, and Salyan districts over the study period. The study also aimed to identify stillbirths; however, high data missingness precluded their inclusion in this analysis. Districts were selected to represent Nepal’s three ecological areas: mountain, hill, and Terai (plains), from among those that had implemented the CB-NCP for at least one year. In each district, IRHDTC identified neonatal deaths for inclusion by reviewing multiple data sources, beginning with CB-NCP reports at the District Health Office (DHO) or District Public Health Office (DPHO). FCHVs were responsible for completing these reports for deaths that occurred outside of a facility as part of their antenatal and post-partum home visits, which ran until the 28th day of life. Data in the CB-NCP database were then verified at the health facility by the IRHDTC team. This primary data collection occurred from September to December 2013.

### Verbal autopsy interviews

IRHDTC developed a verbal autopsy questionnaire to ascertain the cause of neonatal death through a two-day consultative workshop with representatives from MoHP, Family Health Division (FHD), Child Health Division (CHD), and several non-profit organizations. The questionnaire was designed using a standard verbal autopsy methodology described by the World Health Organization (WHO) [13]. The questionnaire included an open-ended narrative and closed-ended questions to collect data on the vital statistics of the deceased newborn, morbidity history of the newborn, maternal demographic characteristics, pregnancy and delivery history, essential newborn care practices, and care seeking behaviors during newborn illness.

Study interviewers, who administered the verbal autopsy questionnaire, were non-clinical public health professionals with a minimum of a bachelor level degree in a health field. Preference was given to interviewers with prior training or experience working with the CB-NCP. Interviewers participated in a four-day training on the verbal autopsy questionnaire, including a field trip to Kavre to pre-test the study tools. Study pediatricians conducted the training, covering the aim and objectives of the study, methodology, data collection procedures and tools, components of the CB-NCP, and research ethics.

After identification of neonatal deaths, interviewers visited households of the deceased newborn to interview the mother or the next of kin available who had spent the longest amount of time with the baby during illness and prior to death (Additional file 1). Local community health workers joined interviewers to facilitate the interview and serve as translators. Field coordinators reviewed data for 10% of the neonatal deaths to check for accuracy, completeness, and consistency. Interviews were conducted under the direction of the primary investigator of the original study, a pediatrician from IRHDTC (JRD).

### Cause of death assignment

IRHDTC conducted the cause of death assignment during the original study, after completion of primary data collection and prior to the start of this secondary analysis. A single cause of death was independently assigned for each case by two pediatricians in the IRHDTC research team using a modified version of the Neonatal and Intrauterine Death Classification according to Etiology (NICE) methodology [16]. In the event of conflict in the determination of classification of a death, the two pediatricians attempted to resolve the issue through discussion. If no consensus could be reached, a final determination of classification was made by a third physician, the principal investigator (JRD).

### Statistical analysis

In this secondary analysis, we explored relationships between cause of neonatal death and age of death and risk factors among a sample of live born newborns who died within 28 days of life. Determinants were categorized as: maternal demographic characteristics, antenatal health care utilization, pregnancy and delivery characteristics, and newborn care practices and care seeking practices during newborn illness. We conducted bivariate analyses, reporting numbers and percentages, and assessed differences between cause and age of death groups and participant characteristics using chi-squared tests. Associations between age of newborn death and risk factors were displayed in Kaplan-Meier graphs and evaluated using log-rank tests. Factors associated with cause of death and age of death in bivariate analyses (*p* < 0.05) with < 15% missing data, as well as other factors known from the literature to be risk factors for these outcomes, were included in multivariable regression modeling. In cases where two similar factors met these criteria (e.g., ANC visit attendance and tetanus immunization), one was selected for inclusion in the regression modeling. Separate multinomial logistic regression models were used to estimate relative risk ratios and 95% confidence intervals (CIs) between these factors and the two outcomes of interest: cause and age of death. As a non-death reference group was not available in these data, the sepsis cause of death was selected to serve as the reference category for these analyses because it had the largest number of cases. For the purpose of making general comparisons, we included data from Nepal 2016 Demographic and Health Surveys (DHS) where available [17]. Analyses were conducted in Stata version 14.2.

## Results

**Fig. 1:**
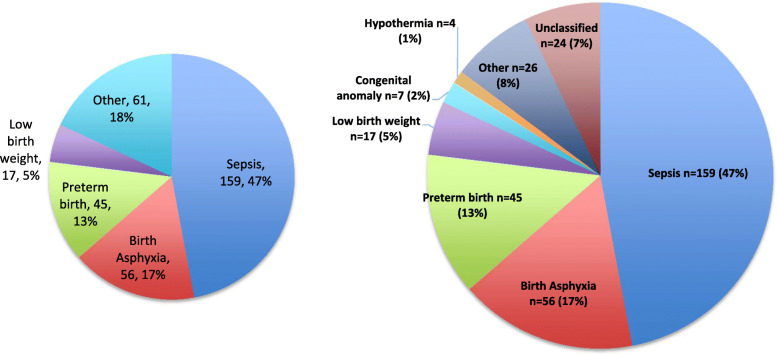
Cause of death distribution for newborns (*n* = 338) in six districts of Nepal from April 2012 to April 2013. Number and percent of deaths by cause for newborns (*n* = 338) in six districts of Nepal from April 2012 to April 2013. The other category included respiratory distress syndrome (*n* = 6, 1.8%), meconium aspiration syndrome (*n* = 5, 1.5%), birth injury (*n* = 2, 0.6%), severe jaundice (*n* = 1, 0.3%), and others (*n* = 12, 3.6%)

Causes of death for 338 newborns, as assigned by the original verbal autopsy study team, are presented in Fig. 1. A quarter (*n* = 89, 26.3%) of neonatal deaths occurred among babies born preterm (< 37 weeks) (term: *n* = 206, 61.0%; post-term: *n* = 43, 12.7%). Of the deaths with a known birth weight, 39.3% (*n* = 81/206) were low birth weight (< 2500 g). Of the infants that were preterm, 48.3% (n = 43) were assigned a cause of death of “prematurity-related,” and of those that were low birth weight, 18.5% (*n* = 15) were assigned a cause of death of “LBW-related.” Approximately a quarter (*n* = 92, 27.2%) of deaths occurred on the day of birth (day 0), and nearly two-thirds (*n* = 219, 64.8%) occurred in the first week (0 to 6 days). Age of death (overall mean of 6.2 days (interquartile range: 0 to 10 days)) differed statistically significantly (*p* < 0.001) by cause of death, with deaths from birth asphyxia, low birth weight, and preterm birth, occurring earlier than those from sepsis.

### Demographic characteristics

Cause of neonatal death was not significantly different across the six study districts (*p* = 0.321). Infant sex (*n* = 191, 56.5% male) varied by district (*p* = 0.015) (percent male: Dolpa (75.0%), Salyan (73.2%), Morang (59.3%), Jumla (56.8%), Palpa (47.7%), and Chitwan (45.9%)). Infant sex, maternal age, religion, caste, smoking, alcohol use, education, and literacy were not associated with cause of death (Table 1). Cause of death varied significantly by maternal gravidity, with a higher proportion of women never previously pregnant occurring among deaths due to birth asphyxia and a lower proportion among sepsis and prematurity (*p* = 0.032). Of the demographic characteristics, only maternal age was related to age of neonatal death. Younger and older women were observed in higher proportions among deaths that occurred on the day of birth (*p* = 0.011) (Supplement Fig. 1).

**Table 1:** Cause of death by demographic characteristics of mothers and newborns

Characteristic	All	Neonatal sepsis	Birth asphyxia	Prematurity related	LBW related	Others	***p***-value^†^
**Sex**^‡^							
Female	147 (43.5)^*^	63 (39.6)	23 (41.1)	22 (48.9)	9 (52.9)	30 (49.2)	
Male	191 (56.5)	96 (60.4)	33 (58.9)	23 (51.1)	8 (47.1)	31 (50.8)	0.548
**Maternal age (years)**							
< 20	75 (22.2)	28 (17.6)	16 (28.6)	11 (24.4)	4 (23.5)	16 (26.2)	
20–24	133 (39.3)	64 (40.3)	21 (37.5)	23 (51.1)	8 (47.1)	17 (27.9)	
25–34	111 (32.8)	58 (36.5)	17 (30.4)	9 (20.0)	5 (29.4)	22 (36.1)	
≥ 35	19 (5.6)	9 (5.7)	2 (3.6)	2 (4.4)	0 (0.0)	6 (9.8)	0.318
**Education**							
Never attended	91 (35.3)	48 (38.7)	15 (37.5)	12 (35.3)	5 (38.5)	11 (23.4)	
Attended some school	167 (64.7)	76 (61.3)	25 (62.5)	22 (64.7)	8 (61.5)	36 (76.6)	0.450
**Literacy**							
Not able to read	97 (37.6)	50 (40.3)	15 (37.5)	12 (35.3)	9 (69.2)	11 (23.4)	
Able to read part of a sentence	48 (18.6)	24 (19.4)	4 (10.0)	6 (17.6)	1 (7.7)	13 (27.7)	
Able to read whole sentence	113 (43.8)	50 (40.3)	21 (52.5)	16 (47.1)	3 (23.1)	23 (48.9)	0.096
**Gravidity**							
0	157 (46.7)	66 (41.8)	36 (64.3)	16 (35.6)	7 (43.8)	32 (52.5)	
1–2	132 (39.3)	69 (43.7)	12 (21.4)	25 (55.6)	7 (43.8)	19 (31.1)	
≥ 3	47 (14.0)	23 (14.6)	8 (14.3)	4 (8.9)	2 (12.5)	10 (16.4)	**0.032**
**Smoking**							
No smoking	294 (87.0)	135 (84.9)	52 (92.9)	40 (88.9)	15 (88.2)	52 (85.2)	
Smoking	44 (13.0)	24 (15.1)	4 (7.1)	5 (11.1)	2 (11.8)	9 (14.8)	0.619
**Alcohol**							
No alcohol	280 (82.8)	132 (83.0)	47 (83.9)	37 (82.2)	15 (88.2)	49 (80.3)	
Alcohol	58 (17.2)	27 (17.0)	9 (16.1)	8 (17.8)	2 (11.8)	12 (19.7)	0.954

^*^ Data presented as number (percent)

^†^ *P*-values from chi-squared tests between each characteristic and causes of death

^‡^ Variable missingness was as follows: education (*n* = 80/338, 23.7%), literacy (*n* = 80/338, 23.7%), and gravidity (*n* = 2/338, 0.6%). Maternal education and literacy data were only available for infant deaths for which the respondent of the neonatal verbal autopsy survey was the mother (*n* = 258/338, 76.3%). Two participants responded “don’t know” to the question about gravidity

### Antenatal care utilization

Higher proportions of women with fewer ANC visits occurred among deaths due to neonatal sepsis and prematurity, relative to birth asphyxia (*p* = 0.037) (Table 2). A similar pattern was observed for mothers who received < 2 doses of tetanus toxoid among deaths due to sepsis and low birth weight (*p* = 0.006). A higher proportion of women who did not make delivery preparations was seen among deaths due to prematurity or other causes, relative to sepsis (*p* = 0.012). Only preparations for delivery was associated with age of death, with deaths on the day of birth having a higher proportion of mothers who made no delivery preparations (Day 0: *n* = 37/92, 40.2%, Days 1–27: *n* = 70/246, 28.5%) (*p* = 0.039).

**Table 2:** Cause of death by maternal antenatal care utilization and pregnancy and labor and delivery characteristics

Characteristic	All	Neonatal sepsis	Birth asphyxia	Prematurity related	LBW related	Others	DHS comparison group (%)^**+**^	*p*-value^†^
**Maternal antenatal care utilization**								
**ANC visits**^‡^								
No ANC	33 (9.8)^*^	21 (13.2)	2 (3.6)	5 (11.1)	1 (5.9)	4 (6.6)	5.9	
1–3 ANC visits	135 (39.9)	62 (39.0)	16 (28.6)	25 (55.6)	6 (35.3)	26 (42.6)	24.8	
≥ 4 ANC visits	170 (50.3)	76 (47.8)	38 (67.9)	15 (33.3)	10 (58.8)	31 (50.8)	69.4	**0.037**
**Tetanus immunization**								
< 2	124 (42.6)	62 (47.3)	16 (30.2)	24 (63.2)	5 (35.7)	17 (30.9)	34.3	
≥ 2	167 (57.4)	69 (52.7)	37 (69.8)	14 (36.8)	9 (64.3)	38 (69.1)	65.7	**0.006**
**Iron/folic acid supplementation**								
No	9 (3.0)	4 (3.0)	1 (1.9)	2 (5.1)	0 (0.0)	2 (3.6)	9.1	
Yes	290 (97.0)	131 (97.0)	53 (98.1)	37 (94.9)	16 (100.0)	53 (96.4)	90.9	0.841
**Delivery preparations made**								
0	107 (31.7)	41 (25.8)	18 (32.1)	21 (46.7)	2 (11.8)	25 (41.0)	16.2	
≥ 1	231 (68.3)	118 (74.2)	38 (67.9)	24 (53.3)	15 (88.2)	36 (59.0)	83.8	**0.012**
**Maternal pregnancy and labor and delivery characteristics**								
**Single or multiple birth**^‡^								
Single birth	318 (94.1)^*^	154 (96.9)	50 (89.3)	38 (84.4)	16 (94.1)	60 (98.4)	–	
Multiple birth	20 (5.9)	5 (3.1)	6 (10.7)	7 (15.6)	1 (5.9)	1 (1.6)	**–**	**0.007**
**Type of delivery**								
Vaginal delivery	289 (86.8)	138 (87.9)	48 (88.9)	40 (88.9)	13 (81.2)	50 (82.0)	91.0	
Cesarean section	38 (11.4)	17 (10.8)	4 (7.4)	4 (8.9)	3 (18.8)	10 (16.4)	9.0	
Other	6 (1.8)	2 (1.3)	2 (3.7)	1 (2.2)	0 (0.0)	1 (1.6)	–	0.747
**Location of birth**								
Home	148 (43.8)	76 (47.8)	21 (37.5)	17 (37.8)	3 (17.6)	31 (50.8)	41.4	
Health facility	172 (50.9)	79 (49.7)	32 (57.1)	23 (51.1)	12 (70.6)	26 (42.6)	57.3	
Other	18 (5.3)	4 (2.5)	3 (5.4)	5 (11.1)	2 (11.8)	4 (6.6)	1.2	0.080
**Assistance at home delivery**								
No assistance	80 (54.1)	45 (59.2)	7 (33.3)	12 (70.6)	2 (66.7)	14 (45.2)	10.0	
SBA	36 (24.3)	15 (19.7)	8 (38.1)	4 (23.5)	1 (33.3)	8 (25.8)	58.1	
Other	32 (21.6)	16 (21.1)	6 (28.6)	1 (5.9)	0 (0.0)	9 (29.0)	12.4	0.284

^*^ Data presented as number (percent)

^+^ Among women age 15–49 with a live birth in the 5 years preceding the survey, Nepal DHS 2016

^†^ *P* values from chi-squared tests between each characteristic and causes of death

^‡^ Variable missingness for maternal antenatal care utilization was as follows: Tetanus immunization (*n* = 47/338, 13.9%) and iron/folic acid supplementation (*n* = 39/338, 11.5%). Data for tetanus immunization and iron/folic acid supplementation were only available for mothers who received at least one ANC visit (*n* = 305/338, 90.2%). While it is likely that the n = 33/338 (9.8%) women who did not receive ANC also did not receive these three interventions, this was not confirmed by the survey, and, therefore, these values were marked as missing data. Variable missingness for pregnancy and labor and delivery characteristics was as follows: type of delivery (*n* = 5/338, 1.5%) and distance from home to HF (*n* = 51/172, 29.7%). Data for assistance at home delivery were collected only for women who delivered at home (*n* = 148/338, 43.8%)

### Pregnancy and labor and delivery characteristics

Multiple birth was significantly related to cause and age of neonatal death, with a higher proportion occurring among deaths due birth asphyxia and prematurity and lower among sepsis and other causes (*p* = 0.007). Deaths for multiple births (Day 0: *n* = 10/92, 10.9%, Days 1–6: *n* = 7/127, 5.5%, Days 7–27: *n* = 3/119, 2.5%) (*p* = 0.038) and mothers attended during delivery by an SBA or other health worker (Day 0: 16/34, 47.1%, Days 1–6: *n* = 6/41, 14.6%, Days 7–27: 14/41, 34.2%) (*p* = 0.009) were observed in higher proportions on the day of birth. Maternal complications during pregnancy and labor and delivery are reported in Supplement Table 1.

### Newborn care practices and care seeking behaviors

None of the newborn care practices assessed were associated with cause of death (Supplement Table 2). Among care seeking behaviors for newborn illness, receiving advice from a health worker to seek care was more common among deaths due to sepsis and less among prematurity and other causes (*p* = 0.002) (Supplement Table 3). Deaths occurring at a health facility, relative to home or other, were observed in higher proportions among deaths due birth asphyxia, prematurity, or low birth weight, and lower for sepsis (*p* = 0.032). Mothers having received advice to seek care occurred in higher proportions among later deaths (Day 0: *n* = 9/37, 24.3%, Days 1–6: *n* = 45/83, 54.2%, and Days 7–27: *n* = 64/96, 66.7%) (*p* < 0.001).

### Multinomial logistic regression models

Relative risk ratios for causes and age of death are presented in Tables 3 and 4, respectively. Women with no previous pregnancies (aRR 2.58, 95% CI: 1.15, 5.83), ≥4 ANC visits (aRR 2.79, 95% CI: 1.30, 5.99) and multiple births (aRR 5.37, 95% CI: 1.40, 20.58) were more likely to have a child who died from birth asphyxia relative to sepsis. Mothers who made ≥1 delivery preparation (aRR 0.35, 95% CI: 0.16, 0.77) were less likely, and women with a multiple birth (aRR 6.12, 95% CI: 1.68, 22.34) more likely, to have a child who died from prematurity than sepsis. Women who were younger (< 20 vs. ≥20- < 30 years) were less likely to have a death occur between Days 1–6 (aRR 0.35, 95% CI: 0.17, 0.72) or Days 7–28 (aRR 0.34, 95% CI: 0.16, 0.71) relative to Day 0. Women with ≥1 delivery preparation (aRR 2.00, 95% CI: 1.03 3.91) were more likely, and those who had a multiple birth (aRR 0.17, 95% CI: 0.04, 0.67) less likely, to have a child who died between Days 7–28 relative to Day 0.

**Table 3:** Multinomial logistic regression analysis for maternal and newborn characteristics and cause of death^a^

Characteristic (***n*** = 326)	Adjusted relative risk ratios (95% CI)							
	Birth asphyxia		Prematurity	LBW-related			Others	
Age (years)								
< 20	1.58	(0.70–3.61)	1.95	(0.76–5.00)	2.52	(0.60–10.68)	1.48	(0.67–3.29)
≥ 20- < 30	Ref		Ref		Ref		Ref	
≥ 30	1.76	(0.67–4.63)	0.63	(0.21–1.85)	1.55	(0.36–6.76)	1.78	(0.75–4.25)
Education								
No	Ref		Ref		Ref		Ref	
Yes	0.62	(0.27–1.41)	1.44	(0.60–3.45)	0.68	(0.19–2.49)	2.11	(0.94–4.71)
Don’t know	1.33	(0.53–3.31)	1.90	(0.69–5.20)	1.15	(0.24–5.58)	1.79	(0.68–4.71)
Nulligravida								
No	Ref		Ref		Ref		Ref	
Yes	2.58	**(1.15–5.83)**	0.52	(0.22–1.23)	0.68	(0.18–2.58)	1.30	(0.63–2.67)
ANC visits								
< 4	Ref		Ref		Ref		Ref	
≥ 4	2.79	**(1.30–5.99)**	0.57	(0.26–1.28)	1.29	(0.40–4.17)	1.32	(0.66–2.64)
Delivery preparations								
0	Ref		Ref		Ref		Ref	
≥ 1	0.56	(0.26–1.21)	0.35	**(0.16–0.77)**	3.79	(0.45–32.07)	0.50	(0.25–1.01)
Multiple births								
Single	Ref		Ref		Ref		Ref	
Multiple	5.37	**(1.40–20.58)**	6.12	**(1.68–22.34)**	1.64	(0.16–17.00)	0.63	(0.07–5.70)
Birth location								
Home or other	Ref		Ref		Ref		Ref	
Health facility	0.83	(0.37–1.87)	1.28	(0.52–3.12)	1.90	(0.47–7.65)	0.71	(0.33–1.52)
Sex								
Female	Ref		Ref		Ref		Ref	
Male	0.99	(0.51–1.92)	0.67	(0.33–1.35)	0.63	(0.21–1.83)	0.74	(0.40–1.37)
Death location								
Home or other	Ref		Ref		Ref		Ref	
Health facility	1.96	(0.90–4.27)	2.26	(0.94–5.43)	2.12	(0.62–7.30)	1.17	(0.54–2.53)

^a^Reference category: Sepsis

**Table 4:** Multinomial logistic regression analysis for maternal and newborn characteristics and age of death^a^

Characteristic (*n* = 326)	Adjusted relative risk ratios (95% CI)			
	Days 1–6		Days 7–27	
Age (years)				
< 20	0.35	**(0.17–0.72)**	0.34	**(0.16–0.71)**
≥ 20- < 30	Ref		Ref	
≥ 30	0.57	(0.25–1.30)	0.80	(0.35–1.82)
Education				
No	Ref		Ref	
Yes	0.96	(0.48–1.91)	1.47	(0.72–3.01)
Don’t know	0.88	(0.39–1.96)	1.15	(0.50–2.66)
Nulligravida				
No	Ref		Ref	
Yes	0.81	(0.41–1.60)	0.78	(0.39–1.55)
ANC visits				
< 4	Ref		Ref	
≥ 4	0.79	(0.42–1.50)	0.80	(0.42–1.53)
Delivery preparations				
0	Ref		Ref	
≥ 1	1.42	(0.74–2.70)	2.00	**(1.03–3.91)**
Multiple births				
Single	Ref		Ref	
Multiple	0.38	(0.13–1.11)	0.17	**(0.04–0.67)**
Birth location				
Home or other	Ref		Ref	
Health facility	1.75	(0.86–3.56)	0.96	(0.46–1.97)
Sex				
Female	Ref		Ref	
Male	0.78	(0.44–1.37)	0.78	(0.43–1.40)
Death location				
Home or other	Ref		Ref	
Health facility	0.68	(0.34–1.35)	0.59	(0.29–1.21)

^a^Reference category: Day 0

## Discussion

We conducted a secondary analysis of data from a verbal autopsy study to understand how cause and age of death differed by demographic, antenatal, intrapartum, and postnatal care factors among neonatal deaths in six districts of Nepal between April 2012 and April 2013. Leading causes of death were sepsis (47.0%), birth asphyxia (16.6%), and preterm birth (13.3%). A high proportion (26.3%) of neonatal deaths were preterm compared to Nepal’s national average in 2012 (14%), indicating, as expected, that the risk of death was higher among preterm babies than those born full term [18–20].

Verbal autopsy data from Nepal’s 2016 Demographic and Health Survey (NDHS) presented a different picture of neonatal mortality; leading causes were: respiratory and cardiovascular disorders of the perinatal period (31%); complications of pregnancy, labor, and delivery (30%); infection (16%); and congenital malformations and deformations (7%) [17]. Definitive conclusions about these two distributions cannot be drawn because of differences in the cause of death assignment methodology used by the NDHS 2016 (World Health Organization International Classification of Disease (ICD) 10) and this verbal autopsy study (NICE). However, the mortality distribution reported in our analysis may indicate that the true underlying neonatal mortality rate in these six districts is high; in such high mortality settings, deaths related to infection generally represent a larger proportion of total deaths [21, 22]. Alternatively, this could suggest that a large proportion of very early neonatal deaths, which are more likely to occur among preterm and asphyxiated babies, were underreported or misclassified as stillbirths in our study [23].

Age of death among newborns in our analysis was high, and the distribution of deaths over the neonatal period was less right skewed than expected. Only 27.2% of neonatal deaths occurred on the first day of life and 64.8% in the first week. This compares to global averages from 2012 of 36 and 73%, respectively, and 57 and 80% from the NDHS 2016 [17, 24]. Similar to the cause of death results, this suggests either a true high neonatal mortality rate or that the verbal autopsy survey missed, or misclassified as stillbirths, many deaths occurring around the time of birth and early days of life. Age of death differed by cause of death, driven primarily by deaths from sepsis, which tended to occur later. As expected, preterm babies tended to die earlier than full term or post-term babies. A trend is visible in the graph of age of neonatal death by maternal age, with younger and older mothers tending to have newborns who die earlier. Higher risk in the youngest age group raises concern given the large proportion of young mothers in Nepal.

Demographic characteristics, including maternal education, literacy, and district, were unassociated with cause and age of neonatal death. However, the infant sex ratio varied widely by district, and was highest in Dolpa (three-quarters male). Globally, studies have reported a biological survival advantage for girls in the early neonatal period; although in South Asia, evidence has indicated a higher risk of mortality among girls, especially in the late neonatal period, an observation attributed to differences in care seeking and gender preference [25–27]. In this context, variation in the infant sex ratio suggests that female deaths may have been underreported. Maternal gravidity varied by cause of death, with women having never been previously pregnant at higher risk of having a neonatal death due to birth asphyxia, an expected result given young maternal age and nulligravida/nulliparity are risk factors for adverse pregnancy outcomes [28]. This suggests a need to ensure young women and first-time mothers are reached with counselling messages and preventive interventions, especially related to birth preparation and institutional delivery.

Coverage of antenatal care and labor and delivery interventions were low among mothers, especially receiving all four ANC visits, institutional delivery, and skilled attendance at birth. Across Nepal, coverage of these life-saving interventions, although higher than seen in our study population, remain below recommended levels, with only 69% of mothers receiving four or more ANC visits and 58% delivered by a skilled provider in 2016 [17]. Several factors relating to contact with the health system during pregnancy, including ANC visits, were associated with a greater proportion of deaths from birth asphyxia relative to other causes. A likely explanation is reporting bias resultant from an increased likelihood of identifying neonatal deaths due to birth asphyxia and other early causes of death among women who utilized ANC and delivery related services. Alternatively, health workers may have been less likely to refer seemingly healthier pregnant women for ANC. Yet, increased coverage of health services alone may not improve survival if care is not of sufficient quality; a study from the Terai region of Nepal suggested that despite the recent rapid increase in institutional deliveries, human resource allocations, health worker knowledge, and stocking of equipment and supplies may not be keeping pace [29].

Of the pregnancy and labor characteristics, only multiple birth was related to cause of death, with multiple births having a higher proportion of deaths attributed to preterm birth and birth asphyxia, an expected result, as the condition is a known risk factor for these outcomes [30, 31]. No significant relationships were identified for newborn care practices and cause of death. However, within care seeking practices, being advised by a health worker to seek care was associated with higher risk of death from sepsis in a bivariate analysis. This could be a result of referral bias, that is, children with severe illness are more likely to be referred, but may not reach care in time to prevent death. A trend towards a greater proportion of sepsis deaths occurring at home or on the way to the health facility, as compared to at a health facility, was observed in bivariate analyses but not the final regression model. This could suggest either that sepsis is more difficult for mothers and caregivers to recognize relative to other illnesses or individuals with sepsis who reach a health facility are less likely to die from the condition. Other studies have highlighted the importance of delays in care seeking for newborn illness in Nepal, especially inability of caretakers to recognize danger signs, delays in the decision to seek care, delays related to first use of home remedies or drugs from a pharmacy, and overreliance on informal providers [32–34].

The study had limitations. The number of neonatal deaths was likely underestimated, due to underreporting and difficulties with identifying and recording cases in rural communities and sparsely populated areas, potentially biasing the cause of and age of death estimates. Stillbirths, which have common risk factors and causes and are frequently misclassified with neonatal deaths, could not be included in this analysis due to data missingness. Effects of referral and reporting biases are a common weakness for the verbal autopsy study approach, especially in areas without advanced reporting systems for neonatal deaths. Recall bias, associated with a long time period between neonatal death and the interview date, may have contributed to data missingness and quality. The lack of controls or “non-deaths” prevented any individual level comparisons making it difficult to draw strong conclusions about how these factors are related to cause and age of death.

## Conclusion

We investigated how antenatal, intrapartum, and postnatal risk factors differed by cause and age of neonatal death in six districts of Nepal. Increased coverage of preventive antenatal care interventions and counseling are critically needed, especially for young women. Delays in care seeking for newborn illness and quality of care around the time of delivery and for sick newborns are important points of intervention with potential to reduce deaths, particularly for birth asphyxia and sepsis, which remain common in this population.

## Supplementary Information

**Additional file 1: Supplement Table 1.** Maternal complications during pregnancy and labor and delivery. **Supplement Table 2.** Cause of death by newborn care practices. **Supplement Table 3.** Cause of death by care seeking during newborn illness

**Additional file 2: Supplement Fig. 1.** Age of death for newborns (*n* = 338) in six districts of Nepal by various factors. Histogram of age of death for newborns (n = 338) in six districts of Nepal (Panel A) and Kaplan Meier cumulative mortality graphs for age of death by cause of death (Panel B), maternal age (Panel C), and district (Panel D).

## Data Availability

Not applicable.

## Abbreviations

ANC: Antenatal care
CHD: Child Health Division
CB-IMCI: Community-Based Integrated Management of Childhood Illness
CB-NCP: Community-Based Newborn Care Package
CI: Confidence interval
DHO: District Health Office
DPHO: District Public Health Office
FHD: Family Health Division
FCHV: Female Community Health Volunteers
ICD: International Classification of Disease
IRHDTC: Integrated Rural Health Development Training Centre
MoHP: Ministry of Health and Population
NDHS: Nepal Demographic and Health Survey
NICE: Neonatal and Intrauterine Death Classification according to Etiology
SDG: Sustainable Development Goal
WHO: World Health Organization
