# Supplementary material for: Causes and age of neonatal death and associations with maternal and newborn care characteristics in Nepal: a verbal autopsy study
Source: Arch Public Health. 2022 Jan 11;80:26. doi: 10.1186/s13690-021-00771-5 (PMC8751254; doi:10.1186/s13690-021-00771-5)
Supplement: Supplementary file 1 — Additional file 1: Supplement Table 1. Maternal complications during pregnancy and labor and delivery. Supplement Table 2. Cause of death by newborn care practices. Supplement Table 3. Cause of death by care seeking during newborn illness [file 13690_2021_771_MOESM1_ESM.docx]

**Supplement Table 1: Maternal complications during pregnancy and labor and delivery**

| **Characteristic** | | **All** | | **Neonatal sepsis** | | **Birth asphyxia** | | **Prematurity related** | | **LBW related** | | **Others** | | **p-value**^†^ | |  |
| --- | --- | --- | --- | --- | --- | --- | --- | --- | --- | --- | --- | --- | --- | --- | --- | --- |
|  |  |  | |  | |  | |  | |  | |  | |  | |  |
| **Pregnancy complications** | | | | |  | |  | |  | |  | |  | |  | |
| **Antepartum hemorrhage**^‡^ | | | | |  | |  | |  | |  | |  | |  | |
|  | No | | 296 (90.2)^*^ | | 138 (91.4) | | 50 (90.9) | | 37 (82.2) | | 14 (87.5) | | 57 (93.4) | |  | |
|  | Yes | | 32 (9.8) | | 13 (8.6) | | 5 (9.1) | | 8 (17.8) | | 2 (12.5) | | 4 (6.6) | | 0.356 | |
| **Blurred vision** | | |  | |  | |  | |  | |  | |  | |  | |
|  | No | | 239 (73.1) | | 102 (67.5) | | 43 (78.2) | | 34 (77.3) | | 11 (68.8) | | 49 (80.3) | |  | |
|  | Yes | | 88 (26.9) | | 49 (32.5) | | 12 (21.8) | | 10 (22.7) | | 5 (31.2) | | 12 (19.7) | | 0.263 | |
| **Hypertension** | | |  | |  | |  | |  | |  | |  | |  | |
|  | No | | 285 (94.7) | | 138 (97.9) | | 46 (92.0) | | 35 (89.7) | | 13 (100.0) | | 53 (91.4) | |  | |
|  | Yes | | 16 (5.3) | | 3 (2.1) | | 4 (8.0) | | 4 (10.3) | | 0 (0.0) | | 5 (8.6) | | 0.114 | |
| **Convulsions** | | |  | |  | |  | |  | |  | |  | |  | |
|  | No | | 270 (81.8) | | 126 (81.8) | | 44 (80.0) | | 34 (77.3) | | 13 (81.2) | | 53 (86.9) | |  | |
|  | Yes | | 60 (18.2) | | 28 (18.2) | | 11 (20.0) | | 10 (22.7) | | 3 (18.8) | | 8 (13.1) | | 0.774 | |
| **Premature rupture of membranes** | | | | | | |  | |  | |  | |  | |  | |
|  | No | | 217 (89.7) | | 102 (89.5) | | 37 (86.0) | | 31 (88.6) | | 8 (88.9) | | 39 (95.1) | |  | |
|  | Yes | | 25 (10.3) | | 12 (10.5) | | 6 (14.0) | | 4 (11.4) | | 1 (11.1) | | 2 (4.9) | | 0.739 | |
| **Other** | | |  | |  | |  | |  | |  | |  | |  | |
|  | No | | 294 (90.7) | | 133 (89.3) | | 52 (94.5) | | 38 (88.4) | | 15 (88.2) | | 56 (93.3) | |  | |
|  | Yes | | 30 (9.3) | | 16 (10.7) | | 3 (5.5) | | 5 (11.6) | | 2 (11.8) | | 4 (6.7) | | 0.694 | |
|  |  | |  | |  | |  | |  | |  | |  | |  | |
| **Labor/delivery complications** | | | | |  | |  | |  | |  | |  | |  | |
| **Prolonged labor** | | |  | |  | |  | |  | |  | |  | |  | |
|  | No | | 237 (71.2) | | 111 (72.1) | | 37 (66.1) | | 31 (68.9) | | 12 (70.6) | | 46 (75.4) | |  | |
|  | Yes | | 96 (28.8) | | 43 (27.9) | | 19 (33.9) | | 14 (31.1) | | 5 (29.4) | | 15 (24.6) | | 0.840 | |
| **Breech birth** | | |  | |  | |  | |  | |  | |  | |  | |
|  | No | | 324 (97.6) | | 153 (99.4) | | 52 (94.5) | | 44 (97.8) | | 16 (94.1) | | 59 (96.7) | |  | |
|  | Yes | | 8 (2.4) | | 1 (0.6) | | 3 (5.5) | | 1 (2.2) | | 1 (5.9) | | 2 (3.3) | | 0.260 | |
| **Placenta previa** | | |  | |  | |  | |  | |  | |  | |  | |
|  | No | | 329 (99.1) | | 153 (99.4) | | 55 (100.0) | | 45 (100.0) | | 17 (100.0) | | 59 (96.7) | |  | |
|  | Yes | | 3 (0.9) | | 1 (0.6) | | 0 (0.0) | | 0 (0.0) | | 0 (0.0) | | 2 (3.3) | | 0.285 | |
| **Sign of maternal infection after rupture of membranes** | | | | |  | |  | |  | |  | |  | |  | |
|  | No | | 61 (54.5) | | 34 (64.2) | | 7 (43.8) | | 6 (40.0) | | 2 (33.3) | | 12 (54.5) | |  | |
|  | Yes | | 51 (45.5) | | 19 (35.8) | | 9 (56.2) | | 9 (60.0) | | 4 (66.7) | | 10 (45.5) | | 0.278 | |
| **Convulsions** | | |  | |  | |  | |  | |  | |  | |  | |
|  | No | | 312 (94.0) | | 144 (93.5) | | 53 (96.4) | | 43 (95.6) | | 16 (94.1) | | 56 (91.8) | |  | |
|  | Yes | | 20 (6.0) | | 10 (6.5) | | 2 (3.6) | | 2 (4.4) | | 1 (5.9) | | 5 (8.2) | | 0.858 | |
| **Postpartum hemorrhage** | | | | |  | |  | |  | |  | |  | |  | |
|  | No | | 296 (89.2) | | 137 (89.0) | | 51 (92.7) | | 43 (95.6) | | 16 (94.1) | | 49 (80.3) | |  | |
|  | Yes | | 36 (10.8) | | 17 (11.0) | | 4 (7.3) | | 2 (4.4) | | 1 (5.9) | | 12 (19.7) | | 0.092 | |
| **Other** | | |  | |  | |  | |  | |  | |  | |  | |
|  | No | | 312 (94.0) | | 147 (95.5) | | 50 (90.9) | | 42 (93.3) | | 17 (100.0) | | 56 (91.8) | |  | |
|  | Yes | | 20 (6.0) | | 7 (4.5) | | 5 (9.1) | | 3 (6.7) | | 0 (0.0) | | 5 (8.2) | | 0.535 | |

^*^ Data presented as number (percent).

^†^ P values from chi-squared tests between each characteristic and causes of death.

^‡^ Variable missingness was as follows: antepartum hemorrhage (n=10/338, 3.0%), blurred vision (n=11/338, 3.3%), hypertension (n=37/338, 10.9%), convulsions (n=8/338, 2.4%), premature rupture of membranes (n=96/338, 28.4%), pregnancy other (n=14/338, 4.1%); prolonged labor (n=5/338, 1.5%), breech birth (n=6/338, 1.8%), placenta previa (n=6/338, 1.8%), maternal infection (n=226/338, 66.9%), convulsions (n= 6/338, 1.8%), postpartum hemorrhage (n=6/338, 1.8%), and labor and delivery other (n=6/338, 1.8%).

**Supplement Table 2: Cause of death by newborn care practices**

| **Characteristic** | | **All** | | **Neonatal sepsis** | **Birth asphyxia** | **Prematurity related** | **LBW related** | **Others** | **DHS comparison group (%)^+^** | **p-value**^†^ |
| --- | --- | --- | --- | --- | --- | --- | --- | --- | --- | --- |
| **Home deliveries (n=148/338)**  **Delivery attendant hand washing**^‡^ | | |  |  |  |  |  |  |  |  |
|  | No | 12 (12.1)*^*^* | | 6 (12.2) | 0 (0.0) | 2 (16.7) | 0 (0.0) | 4 (21.1) | - |  |
|  | Yes | 87 (87.9) | | 43 (87.8) | 17 (100.0) | 10 (83.3) | 2 (100.0) | 15 (78.9) | - | 0.370 |
| **Clean delivery kit used** | |  | |  |  |  |  |  |  |  |
|  | No | 311 (92.0) | | 150 (94.3) | 48 (85.7) | 43 (95.6) | 17 (100.0) | 53 (86.9) | - |  |
|  | Yes | 27 (8.0) | | 9 (5.7) | 8 (14.3) | 2 (4.4) | 0 (0.0) | 8 (13.1) | - | 0.071 |
| **Cord cutting instrument** | |  | |  |  |  |  |  |  |  |
|  | New blade or boiled blade | 78 (52.7) | | 37 (48.7) | 11 (52.4) | 9 (52.9) | 2 (66.7) | 19 (61.3) | 87.9 |  |
|  | Non-sterile method | 70 (47.3) | | 39 (51.3) | 10 (47.6) | 8 (47.1) | 1 (33.3) | 12 (38.7) | 10.7 | 0.801 |
| **Application to the umbilical stump** | |  | |  |  |  |  |  |  |  |
|  | Oil | 18 (13.8) | | 11 (15.7) | 0 (0.0) | 1 (6.7) | 1 (50.0) | 5 (20.0) | 7.5 |  |
|  | Navi malam~ | 19 (14.6) | | 9 (12.9) | 4 (22.2) | 3 (20.0) | 0 (0.0) | 3 (12.0) | 39.0 |  |
|  | Nothing | 88 (67.7) | | 46 (65.7) | 14 (77.8) | 10 (66.7) | 1 (50.0) | 17 (68.0) | 29.0 |  |
|  | Other | 5 (3.8) | | 4 (5.7) | 0 (0.0) | 1 (6.7) | 0 (0.0) | 0 (0.0) | 22.4 | 0.577 |
| **Skin-to-skin care** | |  | |  |  |  |  |  |  |  |
|  | No | 8 (26.7) | | 3 (20.0) | 1 (16.7) | 0 (0.0) | 0 (0.0) | 4 (66.7) | 37.4 |  |
|  | Yes | 22 (73.3) | | 12 (80.0) | 5 (83.3) | 3 (100.0) | 0 (0.0) | 2 (33.3) | 62.6 | 0.084 |
| **All deliveries**  **Time to first bathing** | |  | |  |  |  |  |  |  |  |
|  | <24 hours | 28 (20.9) | | 25 (24.0) | 1 (25.0) | 1 (16.7) | 0 (0.0) | 1 (5.6) | 28.8 |  |
|  | ≥24 hours | 106 (79.1) | | 79 (76.0) | 3 (75.0) | 5 (83.3) | 2 (100.0) | 17 (94.4) | 70.0 | 0.431 |
| **Time to breastfeeding** | |  | |  |  |  |  |  |  |  |
|  | <1 hour | 187 (81.3) | | 111 (78.7) | 10 (62.5) | 25 (86.2) | 8 (88.9) | 33 (94.3) | 45.1 |  |
|  | ≥1 hour | 43 (18.7) | | 30 (21.3) | 6 (37.5) | 4 (13.8) | 1 (11.1) | 2 (5.7) | 54.9 | 0.061 |
| **Fluids other than breast milk given** | |  | |  |  |  |  |  |  |  |
|  | No | 272 (90.1) | | 130 (87.8) | 45 (97.8) | 37 (94.9) | 12 (85.7) | 48 (87.3) | - |  |
|  | Yes | 30 (9.9) | | 18 (12.2) | 1 (2.2) | 2 (5.1) | 2 (14.3) | 7 (12.7) | - | 0.223 |
| **Time to exam of the baby** | |  | |  |  |  |  |  |  |  |
|  | <24 hours | 144 (90.0) | | 66 (85.7) | 28 (100.0) | 16 (88.9) | 9 (100.0) | 25 (89.3) | 91.5 |  |
|  | ≥24 hours | 16 (10.0) | | 11 (14.3) | 0 (0.0) | 2 (11.1) | 0 (0.0) | 3 (10.7) | 8.5 | 0.221 |

^*^ Data presented as number (percent)

^+^ Among most recent live births in the 2 years preceding the survey, Nepal DHS 2016.

^†^ P values from chi-squared tests between each characteristic and causes of death.

^‡^ Variable missingness was as follows: delivery attendant hand washing (n=49/148, 33.1%), clean delivery kit used (n=18/148, 12.2%), application to umbilical stump (n=18/148, 12.2%), skin-to-skin care (n=118/148, 79.7%), time to first bathing (n=204/338, 60.4%), time to breastfeeding (n=108/338, 32.0%), fluids other than breast milk given (n=36/338, 10.7%), and time to exam of baby (n=178/338, 52.7%). Data for delivery attendant hand washing, clean delivery kit used, cord cutting instrument, application to the umbilical stump, and skin-to-skin care were only collected for women who delivered at home (n=148/338, 43.8%).

~ Only one district (Jumla) had the Navi Malam program implemented at the time of data collection.

**Supplement Table 3: Cause of death by care seeking during newborn illness**

| **Characteristic** | | **All** | **Neonatal sepsis** | **Birth asphyxia** | **Prematurity related** | **LBW related** | **Others** | **DHS comparison group (%)^+^** | **p-value**^†^ |
| --- | --- | --- | --- | --- | --- | --- | --- | --- | --- |
| **Time from illness to death**^‡^ | | |  |  |  |  |  |  |  |
|  | Day 0 | 100 (29.6)^*^ | 8 (5.0) | 35 (62.5) | 23 (51.1) | 5 (29.4) | 29 (47.5) | 57% |  |
|  | Days 2-6 | 169 (50.0) | 103 (64.8) | 19 (33.9) | 16 (35.6) | 9 (52.9) | 22 (36.1) | 22% |  |
|  | Days 7-27 | 69 (20.4) | 48 (30.2) | 2 (3.6) | 6 (13.3) | 3 (17.6) | 10 (16.4) | 21% | **<0.001** |
| **Time from illness to home treatment** | | | |  |  |  |  |  |  |
|  | Immediately | 41 (19.2) | 20 (16.5) | 10 (41.7) | 4 (15.4) | 1 (16.7) | 6 (16.2) | - |  |
|  | 1-7 days | 108 (50.5) | 67 (55.4) | 8 (33.3) | 12 (46.2) | 3 (50.0) | 18 (48.6) | - |  |
|  | After a week | 6 (2.8) | 6 (5.0) | 0 (0.0) | 0 (0.0) | 0 (0.0) | 0 (0.0) | - |  |
|  | Treatment not done | 59 (27.6) | 28 (23.1) | 6 (25.0) | 10 (38.5) | 2 (33.3) | 13 (35.1) | - | 0.159 |
| **Advised by HW to seek care** | | |  |  |  |  |  |  |  |
|  | No | 98 (45.4) | 42 (33.6) | 16 (55.2) | 15 (62.5) | 3 (75.0) | 22 (64.7) | - |  |
|  | Yes | 118 (54.6) | 83 (66.4) | 13 (44.8) | 9 (37.5) | 1 (25.0) | 12 (35.3) | **-** | **0.002** |
| **Time from illness to care seeking** | | | |  |  |  |  |  |  |
|  | <1 hour | 54 (49.5) | 36 (48.0) | 6 (50.0) | 4 (44.4) | 1 (100.0) | 7 (58.3) | - |  |
|  | 1-2 hours | 41 (37.6) | 30 (40.0) | 5 (41.7) | 3 (33.3) | 0 (0.0) | 3 (25.0) | - |  |
|  | >2 hours | 14 (12.8) | 9 (12.0) | 1 (8.3) | 2 (22.2) | 0 (0.0) | 2 (16.7) | - | 0.935 |
| **Location of care seeking** | | |  |  |  |  |  |  |  |
|  | Hospital | 36 (26.7) | 17 (20.0) | 8 (38.1) | 6 (54.5) | 0 (0.0) | 5 (33.3) | - |  |
|  | Health facility | 43 (31.9) | 27 (31.8) | 8 (38.1) | 2 (18.2) | 2 (66.7) | 4 (26.7) | - |  |
|  | Private clinic | 22 (16.3) | 13 (15.3) | 4 (19.0) | 2 (18.2) | 1 (33.3) | 2 (13.3) | - |  |
|  | Other | 34 (25.2) | 28 (32.9) | 1 (4.8) | 1 (9.1) | 0 (0.0) | 4 (26.7) | - | 0.147 |
| **Location of death** | |  |  |  |  |  |  |  |  |
|  | Home, on the way, other | 214 (63.3) | 110 (69.2) | 29 (51.8) | 26 (57.8) | 7 (41.2) | 42 (68.9) | 70.6 |  |
|  | Health facility | 124 (36.7) | 49 (30.8) | 27 (48.2) | 19 (42.2) | 10 (58.8) | 19 (31.1) | 29.4 | **0.032** |

^*^ Data presented as number (percent)

^+^ Percent distribution of neonatal deaths from Verbal Autopsy Study of 118 neonatal deaths, Nepal DHS 2016.

^†^ P values from chi-squared tests between each characteristic and causes of death.

^‡^ Variable missingness was as follows: time from illness to home treatment (n=80/294, 27.2%), advised by HW to seek care (n=78/294, 26.5%), time from illness to care seeking (n=6/135, 4.4%). Data for time from illness to home treatment and advised by HW to seek care do not include 44 (n=44/338, 13.1%) infants who were delivered at the health facility and died the same day. Data for time for illness to care seeking and location of care seeking are only available for the 135 (n=135/338, 39.9%) women who sought care for their infants.

Additional file 1:

Neonatal deaths were identified in health facility records (n=194, 57.4%), FCHV reports (n=114, 33.7%), and other sources (n=30, 8.9%). The person interviewed was most often the mother (n=258, 76.3%), followed by the grandmother (n=24, 7.1%), father (n=14, 41%), grandfather (n=4, 1.2%), other relatives (n=24, 7.1%), and non-relatives (n=14, 4.1%).
