# Supplementary material for: Causes and age of neonatal death and associations with maternal and newborn care characteristics in Nepal: a verbal autopsy study
Source: Arch Public Health. 2022 Jan 11;80:26. doi: 10.1186/s13690-021-00771-5 (PMC8751254; doi:10.1186/s13690-021-00771-5)
Supplement: Supplementary file 2 — Additional file 2: Supplement Fig. 1. Age of death for newborns (n = 338) in six districts of Nepal by various factors. Histogram of age of death for newborns (n = 338) in six districts of Nepal (Panel A) and Kaplan Meier cumulative mortality graphs for age of death by cause of death (Panel B), maternal age (Panel C), and district (Panel D). [file 13690_2021_771_MOESM2_ESM.docx]

**Supplement Figure 1: Age of death for newborns (n=338) in six districts of Nepal by various factors**

**A: Age of neonatal death B: Age of death by cause of death**

**
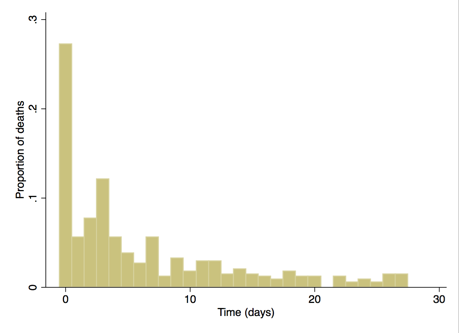

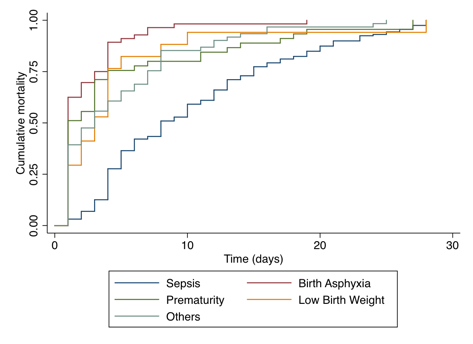
**

*P value for log-rank test <0.001*

**C: Age of death by maternal age D: Age of death by district**


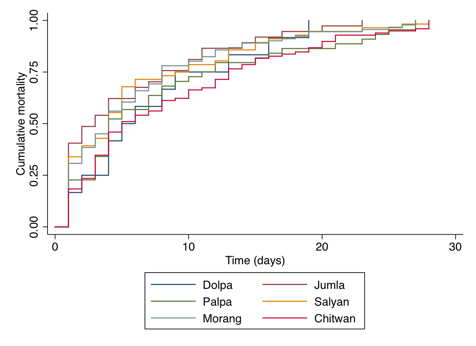
**
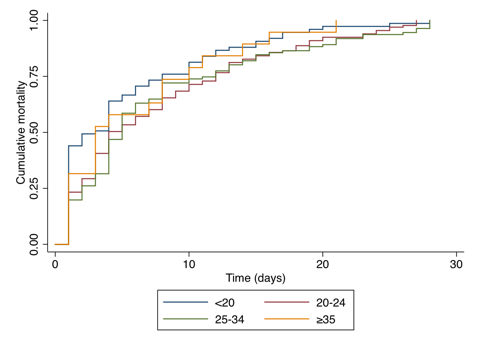
**

*P value for log-rank test = 0.071 P value for log-rank test = 0.086*

**Supplement Figure 1:** Histogram of age of death for newborns (n=338) in six districts of Nepal (Panel A) and Kaplan Meier cumulative mortality graphs for age of death by cause of death (Panel B), maternal age (Panel C), and district (Panel D).
